# Comparison of Climate Change Scenarios of *Rhipicephalus sanguineus* sensu lato (Latreille 1806) from México and the Boarders with Central America and the United States

**DOI:** 10.3390/tropicalmed8060307

**Published:** 2023-06-04

**Authors:** David A. Moo-Llanes, Sokani Sánchez-Montes, Teresa López-Ordoñez, Karla Dzul-Rosado, Daniela Segura-Trejo, Beatriz Salceda-Sánchez, Rogelio Danis-Lozano

**Affiliations:** 1 Centro Regional de Investigación en Salud Pública, Instituto Nacional de Salud Pública, Tapachula 30700, Mexico; david.moo@insp.mx (D.A.M.-L.); tlordonez@insp.mx (T.L.-O.); 2 Centro de Medicina Tropical, Unidad de Investigación en Medicina Experimental, Universidad Nacional Autónoma de México, Ciudad de México 04510, Mexico; sok10108@gmail.com (S.S.-M.); daniela.st@ciencias.unam.mx (D.S.-T.); 3 Facultad de Ciencias Biológicas y Agropecuarias Región Tuxpán, Universidad Veracruzana, Tuxpán de Rodríguez Cano 92870, Mexico; 4 Centro de Investigaciones Regionales Dr. Hideyo Noguchi, Universidad Autónoma de Yucatan, Merida 97000, Mexico; karla.dzul@correo.uady.mx; 5 Laboratorio de Entomología, Instituto de Diagnóstico y Referencia Epidemiológicos, Secretaría de Salud, Ciudad de México 01480, Mexico; betysal_2000@yahoo.com

**Keywords:** ecological niche modeling, México, United States, Central America, *R. sanguineus* sensu lato, climate change

## Abstract

In America, the presence of *Rhipicephalus sanguineus* sensu stricto and *Rhipicephalus linnaei* has been confirmed. Both species are found in sympatry in the southern United States, northern Mexico, southern Brazil, and Argentina. The objective of this work is to evaluate the projection of the potential distribution of the ecological niche of *Rhipicephalus sanguineus* sensu lato in two climate change scenarios in Mexico and the border with Central America and the United States. Initially, a database of personal collections of the authors, GBIF, Institute of Epidemiological Diagnosis and Reference, and scientific articles was built. The ENMs were projected for the current period and two future scenarios: RCP and SSP used for the *kuenm* R package, the ecological niche of *R. sanguineus* s.l. It is distributed throughout the Mexico and Texas (United States), along with the border areas between Central America, Mexico, and the United States. Finally, it is observed that the ecological niche of *R. sanguineus* s.l. in the current period coincides in three degrees with the routes of human migration. Based on this information, and mainly on the flow of migrants from Central America to the United States, the risk of a greater gene flow in this area increases, so the risk relating to this border is a latent point that must be analyzed.

## 1. Introduction

Hard ticks of the Ixodidae family are one of the most relevant groups of ectoparasites for public and veterinary health worldwide. The group comprises 729 described species, of which 283 have been found feeding on humans, and many of them have been implicated in the potential transmission of around 103 species of zoonotic microorganisms [1]. In particular, there is an important species complex which encompasses at least 12 species (previously recognized as the *Rhipicephalus sanguineus* complex) of Afrotropical and Palearctic origin [2].

In the American continent, the presence of two taxa has been confirmed: *Rhipicephalus sanguineus* sensu stricto and the recently proposed *Rhipicephalus linnaei* [3,4]. These two species are found in sympatry in the southern United States, Northern Mexico, Southern Brazil, and Argentina [3]. Until now, the species that describes the largest distribution in the continent was *R. linnaei*, which is found mainly in the Neotropical region and has recently colonized the Southern United States, where it is considered a priority invasive species [5]. Both species are mainly associated with domestic canids that inhabit urban environments, which is why they represent a threat to the health of both companion animals and the human population [6,7]. The two species of ticks are considered vectors of various microorganisms that cause disease in humans (such as *Rickettsia rickettsii* and *Rickettsia massiliae* spotted, the etiological agents of Rocky Mountain Spotted Fever and *R. massiliae* rickettsiosis, respectively), and dogs (*Ehrlichia canis*, *Anaplasma platys* and *Babesia vogelii*, causative agents of canine monocytic ehrlichiosis, thrombocytic anaplasmosis and canine babesiosis, respectively) [8]. Various studies have demonstrated a clear difference in vector competence, host specificity and anthropophilic habits between both taxa [9], making it imperative to understand their geographic distribution to identify priority areas of tick-borne pathogens circulation. During the last 10 years, the number of studies implementing molecular tools as additional characters for the identification of the populations of both taxa of ticks has increased in across the American continent [3]. Nevertheless, there are vast regions in which it is still unknown if both species coexist in sympatry. Additionally, little is known about the environmental conditions that allow the establishment of members of this complex [10], particularly in Mexico where both species are distributed.

Currently, new technological tools such as the Ecological Niche Model (ENM) are frequently used to analyze and predict spatial patterns and the distribution of vector-borne disease. Multiple abiotic and biotic factors have been associated with the ecological niche; in particular: precipitation, temperature, altitude, latitude, physical barriers, host distributions and abundance [11,12]. Clarke-Crespo et al. [13], mentioned there is a greater proportion of species adapted to tropical ecosystems in México, with the more suitable areas for this genus *Rhipicephalus* in costal ecosystems. The genus *Rhipicephalus* is present in all biogeographic regions. However, there are no endemic species of this genus in the Nearctic and the Neotropical regions, which suggests that this genus was probably introduced to the Americas with companion animals from Eurasia and Africa [13]. Alkishe et al. [14] reported models created in different calibration areas showing high agreement of suitable areas among model predictions from the Eastern United States, Southern Mexico, South America, Europe, North Africa, sub-Saharan countries, Asia and Australia.

Recently, as a complement to the ENM, climate change scenarios (future) have been used. Intergovernmental Panel on Climate Change (IPCC) is constantly working on updating the scenarios [15]. In the Fifth IPCC Report (2014), it was proposed to work with the Representative Concentration Pathways (RCP’s). An RCP is a greenhouse gas concentration (not emissions) trajectory adopted by the IPCC. The pathways describe different climate futures, all of which are considered possible depending on the volume of greenhouse gases emitted in the years to come. Subsequently, in the Sixth IPCC Report (2021), modifications are made to the RCPs, which become Shared Socioeconomic Pathways (SSPs). The SSPs are scenarios of projected socioeconomic global changes up to 2100; that is, they are used to derive greenhouse gas emissions scenarios with different climate policies [15]. *Rhipicephalus sanguineus* s.l. is known to maintain populations under a broad range of conditions: e.g., temperatures of 20−35 °C and humidity of 35–95%, so climatic variables may be important predictors of *R. sanguineus* s.l. distribution, and changes in these variables may significantly affect populations of this species [2,3,13,14].

In Mexico, a recent concern is human migration from South America (migrants are cosmopolitan), traveling through Mexico and having the United States as one’s destination. Above all, there is evidence of *R. sanguineus* s.l., a complex of species of African origin that currently has a cosmopolitan distribution because of its dispersal by human migrations that have transported dogs (the main host) from one continent to another. It has become one of the most common ectoparasites transmitted during the import of these domestic animals in the neotropics [3,16]. For this reason, it is imperative to identify the potential distribution of the members of the *R. sanguineus* complex to strengthen the control measures of acaralogical surveillance programs and establish risk mitigation measures for the transmission of key tick-borne pathogens. Thus, our goal in this study was to compile all the information available from scientific publications, Instituto de Diagnóstico y Referencia Epidemiológicos (INDRE) and the Global Biodiversity Information Facility (GBIF; www.gbif.org) to evaluate the projection of the potential distribution of the ecological niche of *Rhipicephalus sanguineus* s.l. in climate change scenarios in México and borders with Central America and the United States.

## 2. Materials and Methods

- Database for *R. sanguineus* s.l.

We built a database of personal collections of the authors, GBIF (https://doi.org/10.15468/dl.7tffuh), INDRE and scientific articles [3,6,17,18] for México and the United States (California, Arizona, New Mexico and Texas). The points of occurrence correspond mainly to Mexico, although we chose four states in the United States due to the effect that the northern border of Mexico may have. In the case of the southern border, for Guatemala and Belize, we did not find occurrences of *R. sanguineus* s.l. A total of 1459 unique occurrence sites were included in the database for *R. sanguineus* s.l. (Table S1), while duplicate occurrences were eliminated to reduce the effects of spatial autocorrelation by thinning records within 5 km of individual occurrence points (N = 430 datapoints) using the *spThin* R package [19].

- Accessible M region

The accessible M region [20] was extended using a 100 km radius buffer around each occurrence point and was subsequently overlaid on the ecoregion shapefile of World Wildlife Fund [21] based on the methodology proposed by Moo-Llanes et al., [12] to avoid a potential modeling bias related to model calibration. The accessible M region is a critical determinant of the outcome of model calibration, model evaluation, and model comparison [18]. We randomly split the occurrence records into two subsets: 70% of occurrences for model calibration and 30% of occurrences for internal testing [22,23] using the “random k-fold” method. The latter method partitions the occurrence localities randomly into a user-specified number of (k) bins, as described by Muscarella et al. [24].

- Bioclimatic Layers: current and future

Fifteen bioclimatic layers (1970–2000) were used to construct ENMs from the WorldClim database version 2.0 [25] (http://www.worldclim.org accessed on 01 December, 2022), excluding those bioclimatic variables (Bio8, Bio9, Bio18 and Bio19) since they show spatial anomalies in the form of odd discontinuities between neighboring pixels [23,26]. All variables had a spatial resolution of 1 km^2^. We used four sets of environmental predictors (Table 1). Set1: 15 bioclimatic variables from WorldClim [23,27]. Set2: variables used for the construction of niche models of other species of medical importance such as *Lutzomyia* spp. and *Brumptomyia* spp. [11], *Aedes* spp., *Anopheles* spp., *Culex* spp., and others [28], and the *Triatoma* spp. [27,29]. Set3: jackknife processes were used in MaxEnt alongside correlation analyses to select the distinct sets of variables that contributed most to the models (>90%), eliminating one variable per pair with the Pearson correlation (r < 0.8). To select the variable that was eliminated, it was verified that the one that presented a lower spatial autocorrelation with the pair of variables to be evaluated, and with other combinations of bioclimatic variables, was selected [11,22,23,27]. Finally, Set4 used the variance inflation factor (VIF) [30], which is a measure of levels of multicollinearity between pairs of variables in the *usdm* R package. Values of VIF > 10 denote a potentially problematic correlation between covariates, indicating that these covariates should be carefully evaluated in model development [23,27].

The ENMs were projected for the time period (1970–2000) and two climate change scenarios: Representative Concentration Pathways (RCP’s) for the year 2050 and Shared Socioeconomic Pathways (SSP’s) for the year 2041–2060. ENMs were projected using the four RCP’s from the Fifth Assessment Report (AR5), representing the lowest to highest estimated greenhouse gas emissions: RCP2.6 (>430 ppm CO_2_), RCP4.5 (580–720 ppm CO_2_), RCP6.0 (720–1000 ppm CO_2_), and RCP8.5 (>1000 ppm CO_2_) and the four SSP’s from the Six Assessment Report (AR6): SSP126 (445.6 ppm CO_2_), SSP245 (602.8 ppm CO_2_), SSP370 (867.2 ppm CO_2_), and SSP585 (1135.2 ppm CO_2_) [15,23]. We use the RCP’s and SSP’s because the former primarily focuses on estimating greenhouse gases emissions, while the second also adds socioeconomic issues for the implementation of public policies.

- Ecological niche modeling for *R. sanguineus* s.l.

The ENM was modeled using the MaxEnt algorithm based on the *kuenm* R package [31,32]. We created candidate models by combining four sets of environmental variables, ten values of regularization multiplier (1–10 with intervals of 1), and seven possible combinations of three feature classes (linear = l, quadratic = q, and product = p) [31]. The model performance and best candidate models were selected first based on statistical significance: partial Receiver Operating Characteristic (partial ROC) and omission rates 5% for predictive ability; and second by performance based on Akaike Information Criterion corrected (AICc) for small sample sizes [33]. The number of parameters is measured simply by counting all parameters with a nonzero weight in the lambda file produced by MaxEnt, a small text file containing model details that MaxEnt produces as part of the modeling process [33]. We selected models with delta AICc ≤ 2 from those that were statistically significant and had omission rates below 5% [31]. After model calibration, we created final models with the selected parameter values, using all occurrences after the corresponding thinning process, with 10 bootstrap replicates with logistic outputs. We created a consensus model with the replicates obtained for each parametrization (continuous) in the step “Final Model construction and evaluation” in *kuenm* R package. The results of the ENM for each scenario were converted into binary maps utilized the projected environmental suitability in geographic space, using the lowest training presence threshold approach; an acceptable error rate of omission of *E* = 5% or 10% was selected (identified the highest suitability threshold that includes [100 − *E*] % of the calibration data [23,27]. Since this package allows the creation of suites of models with multiple sets of parameters, considering all of them in concert will improve the quality and robustness of the predictions. *kuenm* R package allows users to test distinct sets of environmental variables, which can be used to test hypotheses of variable contribution, or to test among distinct calibration areas [31].

- ENM of *R. sanguineus* s.l and migration

We compared the coverage of the binary map in the different scenarios of *R. sanguineus* s.l in the number of pixels of 1 km^2^. The coverage of the current period was calculated, adding the coverage in each of the climate change scenarios. This calculation is called “conserved coverage”; that is, the number of pixels that are maintained from the current period and in the different climate change scenarios. Subsequently, the “gained coverage” was calculated; that is, the number of new pixels in the different climate change scenarios in relation to the current period; and finally, the “lost coverage”; that is, the number of pixels lost from the current period that are not found in the different climate change scenarios. We use the current ENM of *R. sanguineus* s.l to overlap it with the human migration routes that circulate in Mexico, coming from Central America to the United States. The current ENM of *R. sanguineus* s.l. was divided into three risk categories: low (0.15–0.38), medium (0.39–0.52) and high (0.53–0.86); and later, it was to cut it to the routes of human migration in Mexico. The human migration shapefile was built with information from the Comisión Mexicana de Ayuda a Refugiados [34].

## 3. Results

A total of 280 candidate models were built with four sets of predictors for *R. sanguineus* s.l. Model performance occurred under optimal parameters using set environmental predictors (Set3), statistically significant models (N = 209), best candidate models (N = 1), a regularization multiplier (N = 2), features classes (linear), a partial ROC (N = 1.09, a 5% omission rate (N = 0.07), AICc (N = 9979.52), delta AICc (N = 0.00), and number parameters other than 0 that provide information for the construction of the model based on lambdas (N = 6) for *R. sanguineus* s.l.

**Figure 1:**
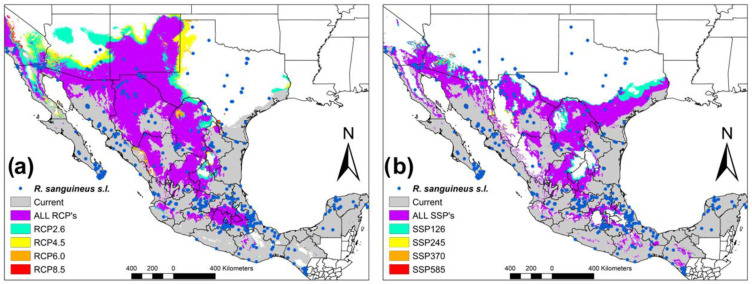
Climate change scenarios of *R. sanguineus* s.l. in México. (**a**) Map where the current period is observed and the different RCP scenarios are added. (**b**) Map where the Current period in observed and the different SSP scenarios are added.

The ecological niche of *R. sanguineus* s.l. is distributed throughout the country, together with border areas between Central America, Mexico, and the United States. The areas with potential distribution are located in the Yucatan Peninsula, all the states of the Pacific coast, Baja California Sur, Baja California Norte, as well as the slope of the states of the Gulf of Mexico, Chihuahua, Durango, and Texas (United States) (Figure 1). It is noteworthy to mention that all potential areas without the presence of *R. sanguineus* s.l. in the present period are occupied by the overlap of the four RCP’s. These areas include Central Mexico, Chihuahua, Durango, Coahuila, as well as the northern border states with the United States. (Figure 1a). In the case of the SSP’s scenarios, the potential coverage is similar, overlapping the RCP’s but including the eastern part of Texas (United States) (Figure 1b).

**Figure 2:**
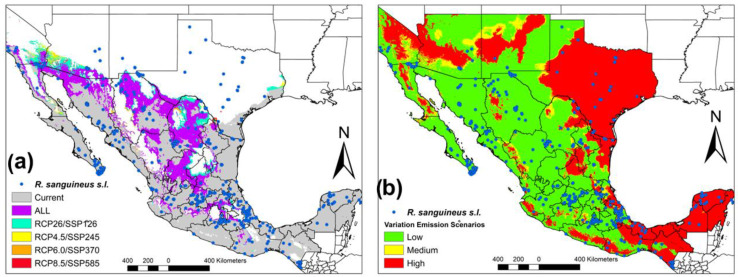
Ecological overlap and variation of the ecological niche patterns of the different RCPs and SSPs in *R. sanguineus* s.l. in Mexico. (**a**) Map of the current ecological niche and overlap by climate change scenarios. (**b**) Map of the variation emissions of climate change scenarios.

Subsequently, the first RCP (RCP2.6) overlaps with its similar SSP (SSP126) and does so successively in all the scenarios. We observe that the geographic overlap of all the scenarios (RCP’s and SSP’s) occurs mainly in Northern Mexico (Sonora, Chihuahua, Coahuila, Durango and Zacatecas), and Center of Mexico (Oaxaca, Puebla, San Luis, and Jalisco) in comparison with the changes in the different scenarios in California, Arizona and Texas (United States) and Coahuila (México) (Figure 2a). When analyzing the variation between all the scenarios in three degrees of variation (low, medium and high), we can see that the lowest variation almost corresponds to the current distribution of *R. sanguineus* s.l. in Mexico, California, Arizona and New Mexico (United States) in comparison with the increase in the variation value corresponding to Yucatan Peninsula, Oaxaca, Tabasco, Chiapas, Pacific Coast and Texas (United States) (Figure 2b).

The highest percentage of conserved coverage corresponds to SSP245 (63.40%) and SSP370 (62.78%), while the highest percentage of gained coverage corresponds to SSP’s (100.00%) and the lost coverage percentage corresponds to RCP6.0 (23.44%). When comparing the consensus maps, the percentage of conserved coverage corresponds to RCP2.6/SSP126 (54.56%), the gained coverage to RCP8.5/SSP585 (64.48%) and lost coverage to RCP2.6/SSP126 (40.87%) (Table 2).

**Figure 3:**
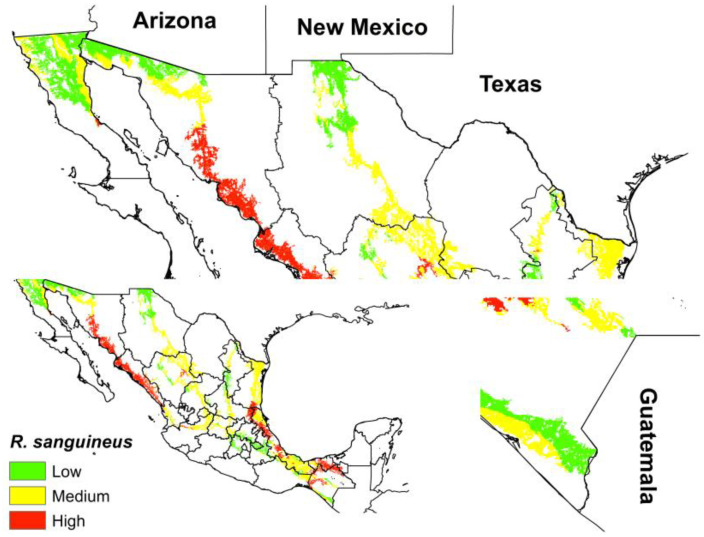
Overlap of the current ecological niche of *R. sanguineus* s.l. with the main routes of human migration in Mexico.

Finally, we can observe that the ecological niche of *R. sanguineus* s.l. for México in the current period coincides with three risk categories with the human migration routes. The highest risk is observed in Northern Chiapas, the Gulf of Mexico, and Sinaloa and Sonora. On the Southern Border, the low category corresponds to the municipalities of Tuxtla Chico, Cacahoatán and Union Juarez that belong to the State of Chiapas, Mexico. While on the Northern Border, the low–medium risk points correspond to San Diego (California), Imperial (California), Yuma (Arizona), Pima (Arizona), Santa Cruz (Arizona), Luna (New Mexico), Dona Ana (New Mexico), El Paso (Texas), Webb (Texas), Hidalgo (Texas) and Cameron (Texas) (Figure 3).

## 4. Discussion

This is an exclusive study which is the first to address the potential distribution of *R. sanguineus* s.l. with emphasis on Mexico. This work results from active acarological surveillance at the national level (Health Secretary), together with data from multiple regional studies, and publications which allow us to establish a much clearer picture of the situation of the *R. sanguineus* complex. There are other articles that address the potential distribution for the genus *Rhipicephalus* in México [13], for *R. sanguineus* s.l. worldwide [32] and the most recent, the ecological niche of Tropical lineage *R. sanguineus* s.l. in the United States [5]. Finally, Sánchez-Pérez et al. [35], assessed the potential effects of climate change on the distribution of *R. sanguineus* in the Americas in 2050 and 2070 using the general circulation model CanESM5 and two SSPs: SSP245 (moderate emissions) and SSP585 (high emissions).

The most complete study based on the terms of ecological niche construction and model uncertainty was carried out by Alkishe et al. [32]. This study used the tick species *R. sanguineus* s.l. (distributed in different areas around the world) to characterize its global geographic distribution using ecological niche modeling and explore the uncertainty involved in transferring models in space and time. In Alkishe et al. [32] used around 368 unique datapoints after data cleaning. We use 430 unique datapoints only for Mexico and the border with the United States, and Pascoe et al. [5] used 593 datapoints for America. Finally, Clarke-Crespo et al. [13] made a first attempt to build the ecological niche of the genus *Rhipicephalus* for Mexico; however, they had the limitation of not being able to make the separations at the species level (only genera). They used 71 datapoints at the generic level for Mexico [13]. Then, Sánchez-Pérez et al. [35] used a total of 335 occurrence points of *R. sanguineus* for America. Therefore, having a more complete picture helps to us make better interpretations of results. Partial ENMs portray range contractions, or under-prediction, at the artificial boundaries and have different patterns of predicted presence and absence. Finally, it is advisable that ENMs use presence data from the complete distribution ranges of species. Furthermore, it should be kept in mind that any ENM essentially has a partial extent in space and time [36]. We used the methodology to mitigate the bias in the use of environmental variables (spatial bias in the variables) (different sets of variables), the parameters in the construction of the ENM (*kuenm* R package) and finally, *spThin* R package mitigated the spatial bias in the occurrences or agglomeration of occurrences in a particular area [19.24]. Unlike mechanistic models, correlative models use global variables (for example: bioclimatic variables based on temperature and precipitation) to explain the potential distribution of a particular species [21,31].

Another similarity between both studies [32] is that the bioclimatic variables that were repeated were temperature seasonality (Bio04), mean temperature of warmest quarter (Bio10) and precipitation seasonality (Bio12). Pascoe et al. [5] used the following bioclimatic variables: Bio04, mean temperature of coldest quarter (Bio11) and precipitation of driest month (Bio14). Finally, Clarke-Crespo et al. [13] used the annual mean temperature (Bio01), Bio04, Bio14, precipitation seasonality (Bio15) and precipitation of the wettest quarter (Bio16) for construction of the ENM. Then, Sánchez-Pérez et al. [35] used the bioclim variables: Bio10, Bio14, and Bio15. It is important to highlight that the variables that provide information for the construction of the ecological niche model have been repeated in various studies. Recent studies have demonstrated that ticks exposed to high temperatures attach and feed on humans and rabbits more rapidly. This observation suggests that the risk of human parasitism by *R. sanguineus* could increase in areas experiencing warmer and/or longer summers, consequently increasing the risk of transmission of zoonotic agents (e.g., *Rickettsia conorii* and *R. rickettsii*) [37]. On the contrary, global warming might prompt the establishment of tick populations in previously free areas. For instance, it has been speculated that an increase of about 2–3 °C in the mean temperature from April to September could result in the establishment of populations of *R. sanguineus* complex in regions of northern temperate Europe [37]. However, the actual impact of global warming on *R. sanguineus* s.l. ticks is uncertain. This should not be left aside when we analyze the impact of *R. sanguineus* s.s. and *R. linnaei.*

The ENM of the genus *Rhipicephalus* for Mexico presents a few useful data [13], emphasizing the need to create a complete database for Mexico and reinforcing the need for records at the sub-species level (*R. sanguineus* s.s. and *R. linnaei*). It is important to note that in previous studies in Mexico using the amplification and sequencing of the mitochondrial 16S-rDNA gene, it has been shown that *R. linnaei* is the most widely distributed species in the country, described in 10 states of the Neotropical region, in contrast with *R. sanguineus* s.s. which is restricted to the Nearctic states of Sonora and Chihuahua [3]. This is consistent with data obtained in Arizona, which show that *R. sanguineus* s.s. limits its zone of sympatry with *R. sanguineus* s.s. in a couple of Mexican states. Since in the present work, only the morphological records prior to the generation of the taxonomic keys of Nava et al. [2] and Slapeta et al. [4] are available, it is not possible to guarantee that all the records in this work correspond exclusively to one species or another. However, given the marked behavior that both species have shown, it is possible to assume that they are in sympatry only in the Nearctic states, a hypothesis that should be tested by implementing and generating more extensive studies at the regional level. Unlike the study by Sánchez-Pérez et al. [35], we have further defined the regions of Mexico, where the presence of *R. sanguineus* in Mexico is projected. This may be due to building the ecological niche with all the variables taking into account this complex species. It is very important to highlight the need to be able to define the limits within the species complex, taking into account ecological, genetic, and morphological issues. An essential point in the construction of the ecological niche is the feasibility of the *kuenm* R package to be able to establish different sets of variables and to be able to evaluate the best model among all of them. This was one of the limitations of the study by Sánchez-Pérez et al. [35]. In the same way, we once again highlight the need to carry out a study at the American level, where we can evaluate the limits between the species that make up the *R. sanguineus* complex based on ecological, genetic and morphological issues but from the point in view of using the same individuals to assess those aspects. Finally, something that has not continued is a review of the areas where the potential distribution exists to find out if the report is true positive or false positive. This information could be useful to validate the ENM used.

Our results of the ENM of RCP4.5 and RCP8.5 are similar to those proposed by Alkishe et al. [32]. However, these scenarios were proposed in the Fifth Intergovernmental Panel on Climate Change Report in 2014. Currently, the new ENMs are being built with the scenarios proposed in the Sixth IPCC Report published in 2021, which have additional socioeconomic characteristics [15]. Recently, they have used the SSPs scenarios to project the ecological niche of *R. sanguineus* s.l. in the Americas, with emphasis on the Southeastern United States [5]. It is important to mention that the SSPs scenarios present more defined risk control regions for the presence of *R. sanguineus* in Mexico and the border with the United States. When we evaluate the RCP’s and SSP’s scenarios, we can observe that there are areas that overlap between both models, which helps us to have less uncertainty when interpreting the results. In Figure 1a, it can be seen that although the increase in greenhouse gases is low (RCP2.6), it is distributed throughout Mexico. Therefore, evidence of outbreaks of various diseases transmitted by ticks in Mexico remains latent. However, in relation to Figure 1b, this scenario seems more encouraging, as it is the most conservative scenario (SSP126) and SSP370 has an increase towards the north of Mexico. Therefore, in both scenarios, there remains a latent alarm that climate change issues will significantly modify the potential distribution of this vector in Mexico. Something that we have to be clear about is the fact that if we do not start with control measures, acarological surveillance and more studies on the different lineages in America, we cannot understand the problems that we will face later on (Figure 2a). Finally, we can have less uncertainty when analyzing variation in climate change scenarios (Figure 2b) where we can see that the greatest variation in the models corresponds to areas in which the presence of *R. sanguineus* is scarce, except Tabasco, Chiapas, Yucatan Peninsula and Texas (United States). Currently, comparisons have been made between different general circulation models in different climate change scenarios. However, in this paper, we compare the last two IPCC reports (RCP’s and SSP’s) [15]. The consensus maps show conserved areas of approximately 50%; however, it is noteworthy to mention that the areas gained (29.13–64.48%) and the areas lost (35.52–40.87%) are almost similar to the conserved area. Therefore, there are increasing concerns about gains in areas in which it is possible that the transmission cycle may bring modifications that would affect the vector control of *R. sanguineus* s.l. In Alkishe et al. [32], they made comparisons between general circulation models, between replicates, between parameters and between RCP’s, showing variation depending on their objective and the group of *R. sanguineus* complex. Therefore, it is crucial to be able to have an ecological niche based on morphological, genetic and ecological characteristics and to be able to participate in decision making in different areas.

Our results are complementary to what was presented by Pascoe et al. [5] in their project using SSP126 and SSP585 for the Southeastern United States, mainly Texas. This state is one of the access points to the United States. It is worth mentioning that Pascoe et al. [5] conclude that our models indicate that tropical *R. sanguineus* has yet to occupy its entire potential range for current climatic conditions, although there is already evidence for the tick expanding in range. Previous studies have shown that *R. sanguineus* s.s. tends to establish itself in temperate regions in Brazil, Argentina, the United States, and Mexico, so in these four countries, the epidemiology of tick-borne pathogens changes radically compared to countries in South and Central America in which only one of these species circulates. For this reason, the niche model is a priority on a regional and/or local scale [3,4]. Our study highlights the need for continuous surveillance and organized surveillance efforts, which can be informed using our models to monitor a range of tick species of medical and veterinary concern. In addition, we demonstrate the importance of confirming tick identification stringently in order to describe current and future predicted risk of ticks and the pathogens they carry. This highlights the need for active acarological surveillance, in addition to raising the alarm to start control measures for ticks that are carried by migrant caravans.

Recently, Danis-Lozano et al. [18] collected *R. sanguineus* s.l from cattle in five municipalities of Chiapas. These ticks were found to be infected with *R. rickettsii* and *Rickettsia felis* in the municipalities that correspond to the human migration routes (Tónala, Pijijiapan, Mapastepec, Ciudad Hidalgo and Tapachula). Therefore, there is an urgent need to carry out active acarological surveillance on the southern border of Mexico. On the Northern border of México, the state of Arizona is a focal point between both countries. Recently, Brophy et al. [38] found the temperate and tropical lineages were well delineated, with some overlap in the eastern part of the state. In one county, tropical and temperate ticks were collected from the same dog host, demonstrating that the two lineages are living in sympatry in some instances and may co-feed on the same host. This has important implications for public health, since each of these species demonstrates a marked vector competence, with *R. sanguineus* s.s. as a vector of *R. massiliae*, whereas *R. linnaei* is a vector of *R. rickettsii*. For this reason, and given previous findings of both bacterial species across the state of Chihuahua, the outlook for the transmission of a broader range of pathogens increases, for which reason distribution models based on the future are a priority. They will be used to identify risk regions that should be considered for the implementation of differential diagnostic methods for the species of pathogens that both tick specie may be carrying regionally. A clear example is *Haemaphysalis longicornis*, which transmits the pathogen *Theileria orientalis* var. Ikeda. This species is native to Asia; however, it has recently invaded the United States and has the potential to invade Mexico [39].

In recent years, the Comisión Nacional de Ayuda a Refugiados has increased the number of migrants who are carrying out their process in the different offices in Mexico. In the year 2021, approximately 110,000 registered migrants were identified; however, the number of unregistered migrants may be higher [34]. Approximately 80,000 migrants enter the southern border (Tapachula, Chiapas) and continue along the different human migration routes until they reach the different points on the northern border. In addition, the displacement of migrants is a point of alarm for the introduction of invasive species, and ectoparasites that move in caravans, emphasizing that many people come with pets and more recently, when *R. sanguineus* was found in cattle. Finally, based on this information, and mainly on the flow of migrants from Central America to the United States, the risk of a greater genetic flow in this area increases the risk on this border. This is a latent point that must be analyzed. According to Figure 3, the northern border areas (borders with Arizona, New Mexico and Texas) and the southern border (border with Guatemala) present low–medium levels of potential distribution of *R. sanguineus* s.l.; however, these migration routes highlight the latent alarm associated with the displacement of this tick, as it belongs to the migrant caravans that constantly leave Tapachula, Chiapas, looking for its border with the United States.

## 5. Conclusions

This article is one of the first to focus mainly on *R. sanguineus* s.l. for Mexico and the border of the United States. The information collected is the most recent in terms of the construction of the ecological niche. Our results have a high suitability in the large regions of Mexico, and in different scenarios (RCP’s and SSP’s) the increase is greater. The routes of human migration could be areas of movement of ectoparasites throughout the Mexican territory. Therefore, we sound the alarm regarding the need for control measures for active acarological surveillance on the northern and southern borders of Mexico, in order to resolve the challenges as soon as possible.

## Figures and Tables

**Table 1:** Set of bioclimatic variables used for the construction of ENM for *R. sanguineus* s.l.

Set1	Set2	Set3	Set4
Bio1 *, Bio2, Bio3, Bio4, Bio5, Bio6, Bio7, Bio10, Bio11, Bio12, Bio13, Bio14 *, Bio15 *, Bio16 and Bio17	Bio1 *, Bio4, Bio5, Bio6, Bio7, Bio12, Bio13, Bio14 *, and Bio15 *	Bio1 *, Bio2, Bio4, Bio6, Bio7, Bio10, Bio14 *, Bio15 *, Bio16, and Bio17	Bio1 *, Bio2, Bio3, Bio13, Bio14 *, and Bio15 *

Bio1 = Annual Mean Temperature; Bio2 = Mean Diurnal Range; Bio3 = Isothermality; Bio4 = Temperature Seasonality; Bio5 = Maximum Temperature of Warmest Month; Bio6 = Minimum Temperature of Coldest Month; Bio7 = Temperature Annual Range; Bio10 = Mean Temperature of Warmest Quarter; Bio11 = Mean Temperature of Coldest Quarter; Bio12 = Annual Precipitation; Bio13 = Precipitation of Wettest Month; Bio14 = Precipitation of Driest Month; Bio15 = Precipitation Seasonality; Bio16 = Precipitation of Wettest Quarter; and Bio17 = Precipitation of Driest Quarter. * These variables are repeated in the four sets.

**Table 2:** Percentage coverage (km^2^) of the ecological niche modeling of *R. sanguineus* s.l. in climate change scenarios.

Ecological Niche Models	Conserved Coverage (%)	Gained Coverage (%)	Lost Coverage (%)
Current + RCP2.6	34.48	79.90	20.10
Current + RCP4.5	33.00	79.79	20.21
Current + RCP6.0	33.67	76.56	23.44
Current + RCP8.5	33.72	75.56	24.21
Current + SSP126	60.67	100.00	0.00
Current + SSP245	63.40	100.00	0.00
Current + SSP370	62.78	100.00	0.00
Current + SSP285	60.79	100.00	0.00
RCP2.6/SSP126	54.56	59.13	40.87
RCP4.5/SSP245	51.72	59.30	40.70
RCP6.0/SSP370	52.86	61.37	38.63
RCP8.5/SSP585	52.27	64.48	35.52

## Data Availability

Not applicable.

## Supplementary Materials

The following supporting information can be downloaded at: https://www.mdpi.com/article/10.3390/tropicalmed8060307/s1, Table S1: Occurrences of *Rhipicephalus sanguineus* s.l. in México and the boarder the United States.

## References

[1] Guglielmone A.A., Robbins R.G. (2018). Hard Ticks (Acari: Ixodida: Ixodidae) Parasitizing Humans.

[2] Nava S., Beati L., Venzal J.M., Labruna M.B., Szabó M.P.J., Petney T., Saracho-Bottero M.N., Tarragona E.L., Dantas-Torres F., Silva M.M.S. (2018). *Rhipicephalus sanguineus* (Latreille, 1806): Neotype designation, morphological re-description of all parasitic stages and molecular characterization. Ticks Tick-Borne Dis..

[3] Sánchez-Montes S., Salceda-Sánchez B., Bermúdez S.E., Aguilar-Tipacamú G., Ballados-González G.G., Huerta H., Aguilar-Domínguez M., Mora J.D., Licona-Enríquez J.D., Mora D.D. (2021). *Rhipicephalus sanguineus* Complex in the Americas: Systematic, Genetic Diversity, and Geographic Insights. Pathogens.

[4] Šlapeta J., Halliday B., Chandra S., Alanazi A.D., Abdel-Shafy S. (2022). *Rhipicephalus linnaei* (Audouin, 1826) recognised as the “tropical lineage” of the brown dog tick *Rhipicephalus sanguineus* sensu lato: Neotype designation, redescription, and establishment of morphological and molecular reference. Ticks Tick-Borne Dis..

[5] Pascoe E.L., Nava S., Labruna M.B., Paddock C.D., Levin M.L., Marcantonio M., Foley J.E. (2022). Predicting the northward expansion of tropical lineage *Rhipicephalus sanguineus* sensu lato ticks in the United States and its implications for medical and veterinary health. PLoS ONE.

[6] Salceda-Sánchez B., Gasca-Zarate C.M., Jiménez-Soto K., Grostieta E., López-Sánchez C.G., Soto-Gutiérrez J.J., Lammoglia-Villagómez M.Á., Huerta-Peña J., Hernández-Carbajal G.R., Chagoya-Fuentes J.L. (2022). Molecular detection of *Rickettsia felis* in fleas and ticks collected from dogs and cats of Puebla, Mexico. Zoonoses Public Health.

[7] Grant A.N., Lineberry M.W., Sundstrom K.D., Allen K.E., Little S.E. (2023). Geographic distribution and seasonality of Brown Dog Tick Lineages in the United States. J. Med. Entomol..

[8] Lineberry M.W., Grant A.N., Sundstrom K.D., Little S.E., Allen K.E. (2022). Diversity, and geographic distribution of rickettsial agents identified in brown dog ticks from across the United States. Ticks Tick-Borne Dis..

[9] Backus L.H., López Pérez A.M., Foley J.E. (2021). Effect of temperature on Host preference in two lineages of the Brown Dog Tick, *Rhipicephalus sanguineus*. Am. J. Trop. Med. Hyg..

[10] Zemtsova G.E., Apanaskevich D.A., Reeves W.K., Hahn M., Snellgrove A., Levin M.L. (2016). Phylogeography of *Rhipicephalus sanguineus* sensu lato and its relationships with climatic factors. Exp. Appl. Acarol..

[11] Moo-Llanes D.A., Ibarra-Cerdeña C.N., Rebollar-Téllez E.A., Ibáñez-Bernal S., Gonzalez C., Ramsey J.M. (2013). Current and future niche of North and Central American sands flies (Diptera: Psychodidae) in climate change scenarios. PLoS Negl. Trop. Dis..

[12] Moo-Llanes D.A., Pech-May A., Ibarra-Cerdeña C.N., Rebollar-Téllez E.A., Ramsey J.M. (2019). Inferring distributional shifts of epidemiologically important North and Central American sandflies from Pleistocene to future scenarios. Med. Vet. Entomol..

[13] Clarke-Crespo E., Moreno-Arzate C.N., Lopez-Gonzalez C.A. (2020). Ecological niche models of four hard tick genera (Ixodidae) in México. Animals.

[14] Alkishe A., Raghavan R., Peterson A.T. (2021). Likely geographic distributional shifts among medically important tick species and tick-associated diseases under climate change in North America: A review. Insects.

[15] Masson-Delmotte V., Zhai P., Pirani A., Connors S.L., Pean C., Berger S., Caud N., Chen Y., Goldfarb L., Gomis M.I., IPCC (2021). IPCC: 2021. Climate Change 2021. The Physical Science Basic. Contribution of Working Group I to the Sixth Assessment Report of the Intergovernmental Panel on Climate Change.

[16] Walker J.B., Keirans J.E., Horak I.G. (2000). The Genus Rhipicephalus (Acari: Ixodidae). A Guide to the Brown Ticks of the World.

[17] Ulloa-García A., Dzul-Rosado K., Bermúdez S.E., López-López N., Torres-Monzón J.A. (2020). Detección de *Rickettsia typhi* en *Rhipicephalus sanguineus* s.l. y *Amblyomma mixtum* en el sur de México. Salud Publica México.

[18] Danis-Lozano R., Camacho-Ramirez S., Álvarez-Hernández G., Leyva-Gastelum M., Cisneros-Vázquez L.A., Dzul-Rosado K.R., Fernández-Salas I., López-Ordoñez T. (2023). Evidencia molecular de *Rickettsia rickettsii* y *Rickettsia felis* en garrapatas colectadas en ganado bovino en la costa de Chiapas. Salud Publica México.

[19] Ailello-Lammens M.E., Boria R.A., Radosavljevic A., Vilela B., Anderson R.P. (2015). spThin. An R package for spatial thinning of species occurrence records for use in ecological niche models. Ecography.

[20] Barve N., Barve V., Jimenez-Valverde A., Lira-Noriega A., Maher S.P., Peterson A.T., Soberón J., Villalobos F. (2011). The crucial role of the accessible area in ecological niche modeling and species distribution modeling. Ecol. Model..

[21] Olson D.M., Dinestein E., Wikramanayake E., Burgess N.D., Powell G.V.N. (2001). Terrestrial ecoregions of the World: A new map of life on Earth. Bioscience.

[22] Moo-Llanes D.A., Montes de Oca-Aguilar A.C., Rodriguez-Rojas J.J. (2020). Pattern of climate connectivity and equivalent niche of Triatominae species of the *Phyllosoma* complex. Med. Vet. Entomol..

[23] Moo-Llanes D.A., Montes de Oca-Aguilar A.C., Romero-Salas D., Sánchez-Montes S. (2021). Inferring the potential distribution of an emerging rickettiosis in America. Case Rickettsia Park. Pathog..

[24] Muscarella R., Galante P.J., Soley-Guardia M., Boria R., Kass J.M., Uriarte M., Anderson R.P. (2014). ENMeval: An R package for conducting spatially independent evaluations and estimating optimal model complexity for MaxEnt ecological niche models. Methods Ecol. Evol..

[25] Fick S.E., Hijmans R.J. (2017). WorldClim 2: New 1-km spatial resolution climate surfaces for global land areas. Int. J. Climatol..

[26] Escobar L.E., Lira-Noriega A., Medina-Vogel G., Peterson A.T. (2014). Potential for spread of the white-nose fungus (*Pseudogymnoascus destructans*) in the Americas: Use of MaxEnt and NicheA to assure strict model transference. Geospat. Health.

[27] Flores-Lopez C.A., Moo-Llanes D.A., Romero-Figueroa G., Guevara-Carrizalez A., López-Ordoñez T., Casas-Martinez C., Samy A.M. (2022). Potential distributions of the parasite *Trypanosoma cruzi* and its vector *Dipetalogaster maxima* highlight areas at risk of Chagas disease transmission in Baja California Sur, México, under climate change. Med. Vet. Entomol..

[28] Bond J.G., Moo-Llanes D.A., Ortega-Morales A., Marina C.F., Casas-Martinez M. (2020). Diversity and potential distribution of culicids of medical importance of the Yucatan Peninsula, México. Salud Publica México.

[29] Ramsey J.M., Peterson A.T., Carmona-Castro O., Moo-Llanes D.A., Nakazawa Y., Butrick M., Tun-Ku E., de la Cruz-Felix K., Ibarra-Cerdeña C.N. (2015). Atlas of Mexican Triatominae (Reduviidae: Hemiptera) and vector transmission of Chagas disease. Mem. Inst. Oswaldo Cruz.

[30] Estrada-Peña A., Estrada-Sanchez A., Estrada-Sanchez D., de la Fuente J. (2013). Assessing the effect of variables and background selection on the capture of the tick climate niche. Int. J. Health Geogr..

[31] Cobos M., Peterson A.T., Barve N., Osorio-Olvera L. (2019). kuenm: An R package for detailed development of ecological niche models using Maxent. Peer J..

[32] Alkishe A., Cobos M.E., Peterson A.T., Samy A.M. (2020). Recognizing sources of uncertainty in disease vector ecological niche models: An example with the tick *Rhipicephalus sanguineus* sensu lato. Perspect. Ecol. Conserv..

[33] Nuñez-Penichet C., Osorio-Olvera L., Gonzalez V.H., Cobos M.E., Jimenez L., DeRaad D.A., Alkishe A., Contreras-Diaz R., Nava-Bolaños A., Utsumi K. (2021). Geographic potential of the world’s largest hornet, *Vespa mandarinia* Smith (Hymenoptera: Vespidae), worldwide and particularly in North America. Peer J..

[34] Comisión Mexicana de Ayuda a Refugiados (2023). Gobierno de México. https://www.gob.mx/comar/articulos/la-comar-en-numeros-323821?idiom=es.

[35] Sánchez-Pérez M., Feria-Arroyo T.P., Venegas-Barrera C.S., Sosa-Gutierrez C., Torres J., Brown K.A., Gordillo-Perez G. (2023). Predicting the impact of climate change on the distribution of *Rhipicephalus sanguineus* in the Americas. Sustainability.

[36] Raes N. (2012). Partial versus full species distribution models. Nat. Conserv..

[37] Dantas-Torres F. (2010). Biology and ecology of the brown dog tick, *Rhipicephalus sanguineus*. Parasites Vectors.

[38] Brophy M., Riehle M.A., Mastrud N., Ravenscraft A., Adamson J.E., Walker K.R. (2022). Genetic variation in *Rhipicephalus sanguineus* s.l. across Arizona. Int. J. Environ. Res. Public Health.

[39] Raghavan R.K., Barker S.C., Cobos M.E., Barker D., Teo E.J.M., Foley D., Nakao R., Lawrence K., Heath A., Peterson A.T. (2019). Potential spatial distribution of the Newly introduced Long-horned tick, *Haemaphysalis longicornis* in North America. Sci. Rep..

